# Management of Molar Incisor Hypomineralization Under the Brazilian Unified Health System

**DOI:** 10.1111/ipd.70134

**Published:** 2026-08-05

**Authors:** Thais Aguiar Gomes, Roberta Costa Jorge, Vera Mendes Soviero

**Affiliations:** ^1^ Department of Preventive and Community Dentistry, Faculty of Dentistry Rio de Janeiro State University Rio de Janeiro Brazil; ^2^ Dental School University Arthur Sá Earp Neto Petrópolis Brazil

**Keywords:** dentistry, molar hypomineralization, national health strategies, primary health care, unified health system

## Abstract

**Background:**

Molar Incisor Hypomineralization (MIH) is challenging for Pediatric Dentists.

**Aim:**

To assess the perception and experience of dentists from the Brazilian Unified Health System (UHS) regarding clinical management of MIH.

**Design:**

This cross‐sectional study was based on a questionnaire including sociodemographic data, perception and treatment decision, and continuing education. The questionnaire scores and independent variables were tested using Student's *t*‐test, ANOVA, and Poisson regression.

**Results:**

The sample comprised 304 dentists. Among available restorative options in the UHS setting, glass ionomer cement was the first choice for severe MIH (65.1% for case 1; 48.4% for case 2). When asked about their first choice when other options were available, indirect restorations (38.9%) and composite (37.2%) were the most preferred. Only 8.9% had used the Hall Technique, but 52% would use it if available. The scale of perception of MIH as a clinical problem resulted in 22.7 (SD = 5.12), being higher among pediatric dentists (PR: 1.23; 95% CI: 1.12–1.33; *p* < 0.001) and dentists working in the Mountain region of the state (PR: 1.09; 95% CI: 1.001–1.179; *p* < 0.001).

**Conclusion:**

UHS dentists frequently treat patients with MIH, acknowledge its clinical implications, and recognize it as a challenge within the UHS.

## Why This Paper Is Important to Pediatric Dentists

- Highlights key challenges in managing severe MIH and explores treatment decisions within a public oral healthcare service.
- Introduces a questionnaire and scale to assess clinicians' perceptions of MIH as a clinical issue.
- Discusses the use of stainless steel crowns following the Hall Technique principle within the context of a public oral healthcare service.

## 1 Introduction

Molar Incisor Hypomineralization (MIH) is a qualitative defect of dental enamel with multifactorial origin combining systemic insults during amelogenesis and genetic predisposition [1]. It is present on all continents, with a global prevalence of 13.5% [2].

Due to the less mineralized dental enamel, patients with MIH have an increased risk of developing caries and requiring dental interventions [3]. The global burden of dental treatment is significantly impacted by MIH, particularly in low‐ and middle‐income countries where the coverage of dental care for the populations remains limited [4]. In Brazil, most of the population seeks dental treatment through the national Unified Health System (UHS). According to the National Oral Health Policy in Brazil, pediatric dental care is provided within the public health service, primarily in primary care under the UHS. In primary care, dentists work as general practitioners, providing oral care to all ages, from children to the elderly [5]. Required treatments in children with MIH are more complex than for those without MIH. Restorations in hypomineralized first permanent molars often involve cusps and multiple tooth surfaces [6]. The treatment decisions in these cases are challenging. Therefore, it is important to investigate how dentists manage the care of children with MIH in the context of UHS.

Since the most recent recommendations from European Association of Pediatric Dentistry on how manage MIH [1], studies that assessed dentist's perceptions and attitudes toward MIH observed that clinicians worldwide reported seeing a high frequency of MIH patients [7, 8, 9, 10, 11], being aware of MIH and its significant clinical impacts and challenging management [7, 9, 12, 13], and expressed interested in receiving clinical training on MIH [8, 10, 11, 12, 14, 15, 16]. The first choice of restorative treatment for severely affected first permanent molars varied from conservative to more invasive restorations, including glass ionomer cement [9, 10], composite resin [11, 12], and stainless steel crowns [12, 14] or extraction [17]. From the three studies [16, 18, 19] conducted in Brazil, two focused on evaluating the knowledge of dentists on MIH [16, 18] and one on how the dentists managed mild and severe cases [19]. In two of them, the questionnaire was shared on social media, not using any strategy to reach a specific sample or region of the country [18, 19]. The other one focused on the dentists working in the UHS in the city of Teresina, in the state of Piauí [16]. The present study aimed to evaluate the perceptions and experiences of UHS dentists in the State of Rio de Janeiro regarding the clinical management of MIH.

## 2 Materials and Methods

This study was approved by the local Research Ethics Committee (Hospital Universitário Pedro Ernesto—CAAE: 48541921.4.0000.5259). Additionally, approval was obtained from the Oral Health technical area of the State Department of Health of Rio de Janeiro through a letter of consent. Participation was voluntary, and consent was provided by clicking on the questionnaire link.

### 2.1 Study Design and Location

This cross‐sectional study was conducted in the State of Rio de Janeiro, located in Southeast Brazil. The state comprises 92 municipalities, has an estimated population of 17,366,189 inhabitants, and a mean Human Development Index of 0.761 [20].

### 2.2 Study Population

In the state of Rio de Janeiro, public health is organized into nine administrative zones. Currently, 92 municipalities have implemented the family strategy care of the UHS, of which 82 integrated with the oral care team. The study focused on municipalities with a population < 1,000,000 inhabitants. Therefore, 830 dentists working in the family strategy care of the UHS across 80 municipalities were eligible to participate. Dentists who had been associated with the UHS for less than 1 year were excluded, since they were considered unlikely to have gained solid experience with the working process.

### 2.3 Construction and Validation of the Instrument

Based on pertinent literature about the concepts to be approached, the researchers developed a questionnaire with 34 questions organized into three sections: (1) sociodemographic data; (2) perception and treatment decision regarding MIH; and (3) continuing education (Supporting Information).

Once the first version of the questionnaire was finalized, it was sent to four dentists who were not affiliated with the UHS but had prior experience with public oral health and knowledge of its current operational standards in Brazil. At this stage, the dentists were asked to conduct a qualitative analysis of the content, answer options, layout, and terminology. Based on their feedback, some sentences and the order of questions and answer options were slightly modified.

In the second stage, three other dentists, who were affiliated with the UHS but in another state, evaluated the questionnaire quantitatively. For each question, the dentists were asked to score clarity (“Is this question simple to understand?”) and pertinence (“Does this question measure what it proposes?”) based on a 5‐point Likert scale (not at all, a little, medium, a lot, or completely). Then, the content validation index (CVI) was calculated [21]. The average CVI was 0.90 for clarity and 0.93 for pertinence (Supporting Information). Values > 0.80 are considered excellent.

Thirdly, the reliability and internal consistency of the instrument were calculated. Ten dentists who treated children but were not affiliated with the UHS responded to the questionnaire twice, with an interval of 8 days. The average intraclass correlation coefficient (ICC) of the agreement between the first and second responses was 0.887.

### 2.4 Application of the Questionnaire

The survey was conducted from January to February 2022 using Google Forms (Google LLC, Mountain View, CA, USA). The link to the form was sent electronically to the oral health coordinators of each municipality, who then forwarded it to the dentists. To increase the response rate, two reminders were sent within a 4‐week period.

### 2.5 Data Analysis

The data were input into an Excel spreadsheet and subjected to analysis using SPSS (v.25, IBM, NY, USA). Demographic data were described using mean and standard deviation for quantitative variables and percentages for qualitative variables.

The questions in Sections 2 and 3, which were related to the perception of MIH as a clinical problem, were assigned scores from 0 to 2 to measure the degree of perception using the “Scale of perception of MIH as a clinical problem by UHS dentists” (Supporting Information). The scale ranged from 0 to 36. Normal distribution of the data was verified using the Kolmogorov–Smirnov test. Student's *t*‐test was used for dichotomous independent variables, while ANOVA was used for ordinal independent variables with three or more categories to assess differences in the mean score of the perception scale among participants in the bivariate analyses. For the multivariate analysis, the generalized Poisson regression model with robust variance was used to assess the association between independent variables in terms of prevalence ratio at the 95% confidence interval. The multivariate analysis included independent variables that were significant in the bivariate analysis or considered clinically relevant. *p*‐values < 0.05 were considered statistically significant.

## 3 Results

Of the 830 dentists invited to participate, 370 (44.6%) responded to the questionnaire, representing 64% of the municipalities and all nine public health administrative regions of the state. Sixty‐six dentists who had been working at the UHS for less than 1 year were excluded from the analysis. The final sample comprised 304 dentists from 64 municipalities and all nine health zones. Table S1 (Supporting Information) presents the distribution of the dentists according to the public health administrative zones.

Demographic data of the participants are displayed in Table 1. Among the 304 dentists, 212 (69.7%) were female, 110 (36.2%) graduated 11–20 years ago, 257 (84.6%) had a postgraduate degree, 234 (77%) were specialists, and 23 (7.5%) had a master's or PhD. Regarding their specialty, 103 (33.9%) were specialized in general family health, 24 (7.9%) in pediatric dentistry, and 130 (42.7%) in other areas. The duration of work as a dentist at the UHS ranged from 1 to 5 years for 41.4% (126) of the participants.

**TABLE 1:** Demographic characteristics of the study participants (*n* = 304 dentists).

	*n*	%
Gender		
Female	212	69.7
Male	92	30.3
Years since graduation		
≤ 10	89	29.3
11 to 20	110	36.2
> 20	105	34.5
Degree level		
Graduate	47	15.5
Specialist	234	77.0
Master/PhD	23	7.5
Specialty		
No postgraduate degree	47	15.5
General family health	103	33.9
Pediatric dentistry	24	7.9
Other	130	42.7
Years in practice in the UHS		
1 to 5	126	41.4
6 to 10	76	25.0
> 10	102	33.6
Years in the current UHS unit		
≤ 5	191	62.8
6 to 10	54	17.8
> 10	59	19.4

Abbreviation: UHS, unified health system.

In the family strategy care, each health unit is responsible for providing health care to the population living nearby. Among the participating dentists, 20.4% assisted more than 3500 people, 18.4% between 1500 and 2500 people, 17.1% up to 1500 people, and 27.6% were unable to report the number of people assisted by their health unit. Regarding the population size of the municipalities, it ranged from 5417 to 886,917 inhabitants (mean = 104,275; median = 39,336).

Tables 2 and 3 present dentists' perceptions regarding the frequency, impact, and challenges of MIH. From the 304 dentists, 272 (89.4%) reported seeing children in their clinical practice every week. Almost all, 291 (95.7%), had seen children with MIH, and 54 (17.8%) reported treating patients with MIH at least once a month. Over 70% of the dentists agreed that MIH negatively impacts the quality of life of patients when molars are severely affected, and over 80% agreed when incisors present demarcated opacities. More than 65% of the dentists reported difficulties in managing partially erupted first permanent molars and a high frequency of restoration failures when treating children with MIH. One‐third of the participants perceived that the frequency of MIH has increased over the years they have worked as dentists at the UHS.

**TABLE 2:** Dentist's perceptions about how often they see children with MIH and the impact of MIH on quality of life (*n* = 304 dentists).

	Never		Rarely		Relatively often		Often		Very often	
	*n*	%	*n*	%	*n*	%	*n*	%	*n*	%
In your clinical practice as a dentist in the UHS										
How often do you see children?^a^	8	2.6	24	7.9	109	35.9	111	36.5	52	17.1
How often do you seechildren with MIH?^b^	13	4.3	110	36.2	127	41.8	30	9.9	24	7.9

	Strongly disagree		Disagree		Neither agree nor disagree		Agree		Strongly agree	
	*n*	%	*n*	%	*n*	%	*n*	%	*n*	%
How much do you agree that MIH impacts quality of life negatively if										
First permanent molars are severely affected?	3	1.0	37	12.2	39	12.8	161	53.0	64	21.1
Permanent incisors present demarcated opacities?	1	0.3	16	5.3	38	12.5	175	57.6	74	24.3

Abbreviations: MIH, molar incisor hypomineralization; UHS, unified health system.

^a^ Rarely: once a week or less; Relatively often: 2 to 5 children per week; Often: 6 to 10 children per week; Very often: > 10 children per week.

^b^ Rarely: once or less in 6 months; Relatively often: 2 to 5 children in 6 months; Often: once a month; Very often: twice or more per month.

**TABLE 3:** Dentists' perceptions about clinical challenges when treating patients with MIH and about MIH frequency along their practice in the UHS (*n* = 304 dentists).

	Do not know		No		Yes	
	*n*	%	*n*	%	*n*	%
When treating children with MIH in the UHS how often you have problems with						
Child's behavior?	37	12.2	132	43.4	135	44.4
Obtaining good local analgesia?	46	15.1	167	54.9	91	29.9
Restoring partially erupted molars?	25	8.2	78	25.7	201	66.1
Frequent restoration failures?	47	15.5	58	19.1	199	65.5
In your perception, has the frequency of MIH been increased along your practice in the UHS?	56	18.4	152	50.0	96	31.6

Abbreviations: MIH, molar incisor hypomineralization; UHS, unified health system.

Table 4 displays the distribution of the 304 dentists according to their choice of treatment for two clinical cases of severe MIH and one case of mild MIH. When asked to indicate which restorative option they would choose among those available in the UHS, hand‐mixed glass ionomer cement (GIC) was the most recommended for both cases, 65.1% for case 1 and 48.4% for case 2. When asked to indicate their first choice independently of being available in the UHS, the most recommended was the individualized indirect restoration for the first case (38.9%) and composite resin for the second (37.2%). Interestingly, 40 (13.2%) would recommend the stainless steel crown for case 1. Only 27 (8.9%) had experience using the Hall Technique, but 158 (52.0%) would recommend it if the stainless steel crowns were available in the UHS. For the mild case, among the options available in the UHS, topical fluoride, gel (27.3%) or varnish (27.3%), was the most indicated treatment, followed by GIC sealant (22.4%). If the indication did not depend on being available in the UHS, fluoride varnish was chosen by 31.6% of dentists, and the indication for resinous (14.5%) and GIC (28.6%) sealants increased.

**TABLE 4:** Treatment indication for first permanent molars affected by MIH (*n* = 304 dentists).

Restorative treatment options (severe cases)	8‐year‐old, no pulp involvement				7‐year‐old, no pulp involvement				Conservative treatment options (mild case)	7‐year‐old, no post‐eruptive breakdown			
	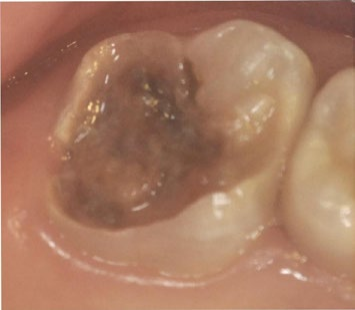				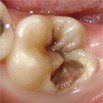					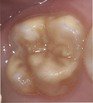			
	Available options at UHS		Free choice of the dentist		Available options at UHS		Free choice of the dentist			Available options at UHS		Free choice of the dentist	
	*n*	%	*n*	%	*n*	%	*n*	%		*n*	%	*n*	%
Amalgam	6	2.0	6	2.0	29	9.5	20	6.6	Monitoring	40	13.2	27	8.9
Hand‐mixed GIC	**198**	**65.1**	59	19.4	**147**	**48.4**	55	18.1	Fluoride gel	**83**	**27.3**	35	11.5
Encapsulated GIC	4	1.3	42	13.9	5	1.6	49	16.1	Fluoride varnish	**83**	**27.3**	**96**	**31.6**
Composite resin	83	27.3	39	12.2	114	37.5	**113**	**37.2**	CPP‐ACP	0	0	13	4.3
Stainless steel crown	0	0	40	13.2	0	0	9	3.0	Resinous sealant	30	9.9	44	14.5
Indirect restoration	9	3.0	**118**	**38.9**	3	1.0	56	18.4	Hand‐mixed GIC sealant	61	20.1	48	15.8
Other	4	1.3	0	0	6	2.0	2	0.7	Encapsulated GIC sealant	7	2.3	39	12.8
									Other	0	0	2	0.7

*Note:* Bold values mean the choice with the highest percentage.

Abbreviations: CPP‐ACP, casein phosphopeptide amorphous calcium phosphate; GIC, glass ionomer cement; MIH, molar incisor hypomineralization; UHS, unified health system.

Most of the dentists reported that they had never accessed educational material about MIH (178; 58.6%), that MIH had never been addressed in the continuing education program at the UHS (243; 79.9%), and that it should be included as a topic in the program (295; 97.0%) (Table S2; Supporting Information). Conservative treatment for molars (257; 84.5%), aesthetic treatment for incisors (223; 73.4%), MIH diagnosis (191; 62.8%), restorative treatment for molars (175; 57.6%), and etiology of MIH (166; 54.6%) were the topics of interest to the dentists (Figure S1; Supporting Information).

The score of perception of MIH as a clinical problem ranged from 10 to 34 with a mean of 22.7 (SD = 5.12) and a median score of 22.5. The variables significantly associated with a greater perception of MIH as a clinical problem were administrative health region (RR = 1.085; CI = 1.002–1.174) and specialty (RR = 1.211; CI = 1.114–1.316) (Table 5). Dentists working in the Mountain region of the state and pediatric dentists reported significantly higher perception of MIH as a relevant clinical problem. Gender was not included in the multivariate analysis because it was highly correlated with the variable specialty. Of the 24 pediatric dentists participating in the study, 22 were women (data not shown).

**TABLE 5:** Bivariate and multivariate analyses of the association between the score of the perception of MIH as a clinical problem and independent variables.

Independent variables	Bivariate analysis^a^				Multivariate analysis^b^			
	*n*	Mean	SD	*p*	Rate Ratio (RR)	Confidence Interval (CI)		*p*
						Min	Max	
Gender								
Male	92	21.7	5.4	0.024				
Female	212	23.2	4.9					
Health region								
Mountain	55	24.0	5.0	0.221	**1.090**	1.005	1.181	**0.036**
North/Northwest	59	22.4	5.1		1.033	0.948	1.124	0.461
Metropolitan I and II	79	22.6	5.7		1.025	0.943	1.115	0.557
Middle south/*Paraíba*	62	22.9	4.3		1.044	0.967	1.129	0.271
*Ilha Grande* Bay/Coastline	49	21.7	5.2		1			
Years since graduation								
> 20 years	105	22.7	5.3	0.672				
11 to 20 years	110	23.0	5.5					
≤ 10 years	89	22.3	4.4					
Degree level								
Master/PhD	23	22.7	5.6	0.660				
Specialist	234	22.9	5.1					
Graduate	47	22.1	4.6					
Specialty								
Pediatric dentistry	24	27.6	5.6	< 0.001	**1.229**	1.132	1.335	**< 0.001**
General family health	103	22.7	4.7		1.000	0.944	1.059	0.990
Other specialties^c^	130	22.1	5.0		1			
Years in practice in the UHS								
> 10	102	23.5	5.4	0.169	1.042	0.982	1.106	0.176
6 to 10	76	22.5	5.3		1.005	0.945	1.069	0.875
1 to 5	126	22.3	4.8		1			
Number of people assisted								
> 1500	168	23.2	5.2	0.076	1.024	0.973	1.077	0.366
≤ 1500	136	22.2	4.9		1			

Abbreviations: MIH, molar incisor hypomineralization; UHS, unified health system.

^a^ Student's *t*‐test for dichotomous data and ANOVA for multi‐categorical data.

^b^ Poisson regression with robust variance.

^c^ Other specialties included those with graduate degrees.

## 4 Discussion

This study collected information on how UHS dentists perceive and manage MIH in their clinical practice. In accordance with previous studies worldwide [7, 8, 9, 10, 11, 14, 19], most dentists reported seeing children with MIH often or very often.

Difficulties in restoring partially erupted molars and a high failure rate of restorations were the main challenges encountered by the dentists when caring for MIH‐affected patients. These challenges are consistent with previous studies reporting difficulties in achieving successful long‐term restorations in hypomineralization teeth [9, 14]. Interestingly, years of clinical experience and degree level were not significantly correlated with the perception of MIH as a challenge. Even the more experienced dentists reported difficulties in treating children with MIH.

Only two factors influenced the dentists' perception of MIH as a clinical problem. It was significantly higher among pediatric dentists and those working in the Mountain region of the state. Previous studies have also observed that pediatric dentists tend to be more aware of the implications of MIH and more confident in diagnosing it compared to general practitioners [8, 11, 12, 14, 15, 18]. In the present study, although pediatric dentists comprised less than 10% of the sample, their training in pediatric dentistry, which supposedly includes MIH, appears to have influenced the results. The variable gender was also significantly associated with the perception scale score in the bivariate analysis, with men presenting a significantly lower mean score. However, gender was not included in the multivariate analysis because it was highly associated with the area of specialty. Of the 24 pediatric dentists participating in the study, 22 were women. We considered that the area of specialty was more relevant in the context of evaluating dentists' perception of MIH than gender itself.

Regarding the region of the state where the dentists worked, those from the Mountain region showed a greater awareness of MIH. In 2018, an epidemiological survey to assess the prevalence of MIH was carried out in the biggest city of the Mountain region [22]. For the study, children were examined at the schools located in the areas covered by the UHS, and the dentists became involved, directly and indirectly, supporting the research team. This would explain why this region was impacted and the dentists showed a greater perception of MIH as an issue in their daily practice.

The decision on which material to use for a restoration is based on several factors. Depending on the cavity shape and extent of the filling, different properties of the dental material may be required. Specifically in MIH, the high protein content in the enamel can compromise the adhesion of resin‐based materials [23], which may influence dentists to prefer GIC as the first choice for direct fillings in accordance with previous studies [9, 10, 14]. However, the mechanical properties of GIC are usually inferior to those of composites [24], which may explain why composites were the first choice in other studies [11, 12]. Indirect restorations, primarily SSC but also individualized onlays, were reported by dentists as the first choice for severely affected first permanent molars in previous studies [12, 14]. It would be expected that indirect restorations would be a common choice of dentists for fillings involving multiple surfaces and cusps. However, treatment decisions also depend on the availability of materials. For this reason, in the present study, the dentists were asked to indicate which restorative option they would use first among the available options at their UHS unit and second, regardless of the availability at their UHS unit.

In the first clinical case, represented by a first permanent molar with widespread post‐eruptive breakdown and caries involving three cusps, the most indicated option changed from hand‐mixed GIC among the available materials at the UHS to individualized indirect restoration when dentists were given a free choice. In the second case, where the extent of the enamel breakdown was smaller but also involved a cusp, hand‐mixed GIC as the first option changed to encapsulated GIC, composite, or indirect restoration when dentists were given a free choice, with composite becoming the most indicated. Interestingly, SSC that had not been indicated by any of the dentists initially was later recommended by 13.2% of them for the first case and by 3% for the second case. These results show that hand‐mixed GIC is the restorative material most used by UHS dentists for severely affected MIH molars, no matter how extensive the filling is. It may explain the high frequency of failures reported by the dentists because direct restorative materials, particularly GIC, might not be the best indication for fillings involving multiple surfaces and cusps. Although the most modern composites have been recommended for the replacement of cusps in the posterior region with an acceptable failure rate, the level of the evidence for this recommendation is very low [25].

Dental treatment provided in primary care under the UHS does not cover indirect restorations. When an indirect restoration is required, the dentist must refer the patient to a specialized unit, where a prosthodontist performs the treatment [5]. However, young children and partially erupted first permanent molars present additional challenges for more complex and time‐consuming procedures such as onlay preparations. Moreover, the demand for prosthetic treatment often exceeds the capacity to accommodate all patients. This explains why individualized indirect restorations were not chosen for most dentists. In this context, SSC could be a more practical alternative as it could be provided at the UHS without referring the patient. However, SSC for permanent molars is not available in the Brazilian market, which explains why it was not selected by any dentists among the available options in the UHS or included among the most recommended options as a free choice. In previous studies, dentists indicated SSC for severely affected MIH molars more frequently than individualized indirect restorations [12, 14].

Over the last 20 years, a simplified technique for using SSC, the Hall Technique, has been extensively evaluated in primary molars and proven to be an effective restorative treatment [26]. In this technique, which is more practical and less time‐consuming, no tooth preparation is required, making local anesthesia unnecessary in most cases. Recently, the Hall Technique has been proposed as an option for treating MIH molars [27, 28], and an ongoing clinical trial is currently evaluating its performance and cost‐effectiveness against GIC restorations [29]. If proven effective also for MIH molars, the Hall Technique could be presented to Brazilian health policymakers as a recommended treatment for children with MIH under the UHS. In the present study, after being introduced to the technique, more than half of the dentists agreed that they would recommend it for severely affected molars if it were an available option.

Topical fluoride was the most frequently selected preventive treatment option, both among the available options at the UHS and as a free choice of the dentist for case 3. However, at least one third of the dentists indicated they would place a sealant, either GIC or resin‐based. This case involved a first permanent molar with yellow‐brownish opacities affecting the buccal, lingual, and occlusal surfaces. Although the available evidence supporting non‐invasive or micro‐invasive treatment for MIH is still limited [30], the treatment decisions of the UHS dentists were aligned with the available recommendations [1].

A strength of the present study is that the questionnaire was carefully designed to assess dentists' perceptions and experience in managing MIH within the context of the UHS. The instrument was validated among experts and demonstrated adequate reliability. More than assessing knowledge, the instrument aimed to quantify how challenging MIH is in the daily practice of the dentists working for the UHS, who are those responsible for delivering primary dental care to most of the population. One limitation of the study is that the survey was restricted to the state of Rio de Janeiro and limited to municipalities with populations under 1 million. However, the response rate of 44.6%, with participation from dentists in 80.0% of the target municipalities, was considered satisfactory. Previous studies reported lower response rates [8, 10, 11]. Nonetheless, it would be valuable to replicate this survey in other regions of the country to gain a more comprehensive understanding of the demands MIH imposes on Brazil's public oral health system.

Only 10% of the dentists reported that MIH had been addressed as a topic in the continuing education program within the UHS. Consistent with previous studies [8, 10, 11, 12, 14, 16, 19], almost all surveyed dentists expressed interest in receiving additional training on MIH. The national protocol guiding oral care implementation within the UHS [5] focuses on major oral health conditions affecting the population, such as dental caries, periodontal disease, oral cancer, dental trauma, malocclusion, edentulism, and dental fluorosis as a developmental defect of enamel, but does not address MIH. According to available official national oral health data, fluorosis affects 16.7% of 12‐year‐old children in Brazil, with over 15% classified as mild or very mild and only less than 2% potentially relevant clinically, indicating it is not a significant public health issue [31]. Given the high frequency of patients with MIH seeking treatment within the UHS and its substantial impact on dental treatment needs [6] greater attention should be given to this condition when formulating guidance documents for dentists working under the UHS.

Dentists working under the Brazilian UHS see children with MIH frequently, perceive MIH as a relevant clinical issue, and identify restoring partially erupted molars and the high failure rate of restorations as the main challenges. Additionally, they recognize that MIH negatively impacts patients' quality of life. Restoration with GIC was the primary treatment option for severely affected molars, although a considerable number of dentists would prefer indirect restorations if they were an available option. Almost all dentists expressed interest in receiving additional training on managing MIH.

## Author Contributions

**Thais Aguiar Gomes:** investigation and data collection. **Roberta Costa Jorge:** writing – original draft and writing – review and editing. **Vera Mendes Soviero:** conceptualization, methodology, investigation, formal analysis, data curation, writing – review, editing and project administration.

## Funding

This work was supported by Fundação de Amparo a Pesquisa do Estado do Rio de Janeiro (SEI‐260003/019626/2022; SEI‐260003/006030/2024; and SEI‐260003/021801/2025) and the Coordenação de Aperfeiçoamento de Pessoal de Nível Superior (001).

## Ethics Statement

This study was approved by the local Research Ethics Committee (HUPE/UERJ ‐ CAAE: 48541921.4.0000.5259). Additionally, approval was obtained from the Oral Health technical area of the State Department of Health of Rio de Janeiro through a letter of consent.

## Consent

Participation was voluntary, and consent was provided by clicking on the questionnaire link.

## Conflicts of Interest

The authors declare no conflicts of interest.

## Supporting information


**Table S1:** Response rate (absolute number and percentage) of municipalities and dentists by public health administrative regions in the state of Rio de Janeiro. Exclusion rate of dentists who had less than 1 year of experience in the Brazilian Unified Health System (UHS) is also shown.
**Table S2:** Data regarding continuing education on MIH and formal registration of MIH cases at the Brazilian Unified Health System (UHS) (*n* = 304 dentists).
**Figure S1:** Proportion of dentists interested in having training on different topics related to MIH (*n* = 304 dentists). English version of the questionnaire is also provided as supplementary material.

## Data Availability

The data that support the findings of this study are available on request from the corresponding author. The data are not publicly available due to privacy or ethical restrictions.
