# Supplementary material for: Management of Molar Incisor Hypomineralization Under the Brazilian Unified Health System
Source: Int J Paediatr Dent. 2026 Aug 5;36(6):866–74. doi: 10.1111/ipd.70134 (PMC13712561; doi:10.1111/ipd.70134)
Supplement: Supplementary file 1 [file IPD-36-866-s001.docx]

Supplementary File

| Table S1: Response rate (absolute number and percentage) of municipalities and dentists by public health administrative regions in the state of Rio de Janeiro. Exclusion rate of dentists who had less than one year of experience in the Brazilian Unified Health System (UHS) is also shown. | | | | | | | | |
| --- | --- | --- | --- | --- | --- | --- | --- | --- |
|  | Municipalities | | | Dentists | | | | |
| Health Regions | Included | Respondents | | Eligible | Respondents | | Excluded | |
|  | N | n | % (n/N) | N | n | % (n/N) | n | % |
| *Ilha Grande* Bay | 3 | 2 | 66.6 | 42 | 9 | 21.4 | 2/9 | 22.2 |
| Coastline | 9 | 8 | 88.8 | 85 | 54 | 63.5 | 12/54 | 22.2 |
| Middle south | 11 | 6 | 54.5 | 103 | 36 | 35.0 | 8/36 | 22.2 |
| *Paraíba* | 11 | 8 | 72.7 | 160 | 39 | 24.4 | 5/39 | 12.8 |
| Metropolitan I | 11 | 10 | 90.9 | 143 | 77 | 53.8 | 20/77 | 26.0 |
| Metropolitan II | 6 | 4 | 66.7 | 52 | 27 | 51.9 | 5/27 | 18.5 |
| Northwest | 12 | 10 | 83.3 | 88 | 42 | 47.7 | 8/42 | 19.0 |
| North | 7 | 7 | 100.0 | 71 | 28 | 39.4 | 3/28 | 10.7 |
| Mountain | 10 | 9 | 90.0 | 86 | 58 | 67.4 | 3/58 | 5.2 |
| Total | 80* | 64 | 80.0 | 830 | 370 | 44.6 | 66/370 | 17.8 |
| * The total includes all municipalities in the state of Rio de Janeiro, except the capital (Rio de Janeiro) and São Gonçalo. | | | | | | | | |

| Table S2: Data regarding continuing education on MIH and formal registration of MIH cases at the Brazilian Unified Health System (UHS) (n = 304 dentists). | | | | | | |
| --- | --- | --- | --- | --- | --- | --- |
| Have you ever assessed educational material on MIH previously through: | Do not know | | No | | Yes | |
|  | n | % | n | % | n | % |
|  |  |  |  |  |  |  |
| lectures, congresses, or other scientific sources? | 1 | 0.3 | 178 | 58.6 | 125 | 41.1 |
| continuing education program at UHS? | 28 | 9.2 | 243 | 79.9 | 33 | 10.9 |
| Would you recommend including MIH as a: |  |  |  |  |  |  |
| topic in the continuing education program at the UHS? | 0 | 0 | 9 | 3.0 | 295 | 97.0 |
| formal entry in patients’ dental records at the UHS? | 51 | 18.8 | 11 | 3.6 | 242 | 79.6 |
| MIH: molar incisor hypomineralization. | | | | | | |

|  | Proportion of dentists (%) |
| --- | --- |
| MIH etiology | 54.6 |
| Restorative treatment for MIH molars | 57.6 |
| MIH diagnosis | 62.8 |
| Esthetic treatment for MIH incisors | 73.4 |
| Conservative treatment for MIH molars | 84.5 |

Figure S1: Proportion of dentists interested in having training on different topics related to MIH (n = 304 dentists).

Box S1 –

English version of the questionnaire.

| SECTION 1 - Sociodemographic data | Scores assigned for analysis*. |
| --- | --- |
| **1.1 Are you female or male?** |  |
| ( ) Female. |  |
| ( ) Male. |  |
| ( ) I prefer not to say. |  |
| **1.2 How many years have you been a dentist? _______________** |  |
| **1.3 What is your level of qualification in dentistry? (you can select more than one option)** |  |
| ( ) Graduation |  |
| ( ) Specialization in Pediatric Dentistry |  |
| ( ) Specialization in Family Health |  |
| ( ) Other specializations |  |
| ( ) Stricto sensu postgraduate courses (Master's or Doctorate) |  |
| **1.4 How long have you worked as a dentist in the Brazilian Unified Health System (UHS)?** |  |
| ( ) Less than 1 year. |  |
| ( ) 1 to 5 years. |  |
| ( ) 6 to 10 years. |  |
| ( ) More than 10 years. |  |
| **1.5 How long have you worked as a dentist in the Brazilian Unified Health System (UHS) with your current Oral Health Team?** |  |
| ( ) Less than 1 year. |  |
| ( ) 1 to 5 years. |  |
| ( ) 6 to 10 years. |  |
| ( ) More than 10 years. |  |
| **1.6- Which health region do you work in in the state of Rio de Janeiro?** |  |
| ( ) *Ilha Grande* Bay (Angra dos Reis, Mangaratiba, Paraty) |  |
| ( ) Coastline (Araruama, Armação dos Búzios, Arraial do Cabo, Cabo Frio, Casimiro de Abreu, Iguaba Grande, Rio das Ostras, São Pedro da Aldeia, Saquarema) |  |
| ( ) Middle south (Areal, Comendador Levy Gasparian, Engenheiro Paulo de Frontin, Mendes, Miguel Pereira, Paracambi, Paraíba do Sul, Paty do Alferes, Sapucaia, Três Rios, Vassouras) |  |
| ( ) *Paraíba (*Barra do Piraí, Barra Mansa, Itatiaia, Pinheiral, Piraí, Porto Real, Quatis, Resende, Rio Claro, Rio das Flores, Valença, Volta Redonda) |  |
| ( ) Metropolitan I (Belford Roxo, Duque de Caxias, Itaguaí, Japeri, Magé, Mesquita, Nilópolis, Nova Iguaçu, Queimados, Rio de Janeiro, São João de Meriti, Seropédica) |  |
| ( ) Metropolitan II (Itaboraí, Maricá, Niterói, Rio Bonito, São Gonçalo, Silva Jardim, Tanguá) |  |
| ( ) Northwest (Aperibé, Bom Jesus de Itabapoana, Cambuci, Cardoso Moreira, Italva, Itaocara, Itaperuna, Laje do Muriaé, Miracema, Natividade, Porciúncula, Santo Antônio de Pádua, São José de Ubá, Varre-Sai) |  |
| ( ) North (Campos dos Goytacazes, Carapebus, Conceição de Macabu, Macaé, Quissamã, São Fidélis, São Francisco de Itabapoana, São João da Barra). |  |
| ( ) Mountain ((Bom Jardim, Cachoeiras de Macacu, Cantagalo, Carmo, Cordeiro, Duas Barras, Guapimirim, Macuco, Nova Friburgo, Petrópolis, Santa Maria Madalena, São José do Vale do Rio Preto, São Sebastião do Alto, Sumidouro, Teresópolis, Trajano de Moraes). |  |
| **1.7- How many inhabitants are there in the municipality where you work as a dentist in the Brazilian Unified Health System (UHS)?** |  |
| ( ) Up to 20,000 inhabitants. |  |
| ( ) > 20,000 and ≤ 40,000 inhabitants. |  |
| ( ) > 40,000 and ≤100,000 inhabitants. |  |
| ( ) >100,000 and ≤400,000 inhabitants. |  |
| ( ) > 400,000 inhabitants. |  |
| ( ) Do not know. |  |
| **1.8- How many users are assisted by your Oral Health Team?** |  |
| ( ) Up to 1500 users. |  |
| ( ) >1500 users and ≤2500 users. |  |
| ( ) >2500 users and ≤3500 users |  |
| ( ) > 3500 users. |  |
| ( ) Do not know. |  |
| **1.9- Is your health unit Modality I or Modality II?** |  |
| ( ) Modality I **(1 Dentist + 1 Assistant or Technician)** |  |
| ( ) Modality II **(1 Dentist + 1 Assistant + 1 Technician)** |  |
| **1.10- In your routine as a dentist in the Brazilian Unified Health System (UHS), how often do you treat children in the dental office?** |  |
| ( ) Rarely (1 child or less per week). |  |
| ( ) Relatively often (up to 5 children a week). |  |
| ( ) Often (6 to 10 children a week). |  |
| ( ) Very often (more than 10 children a week). |  |
| ( ) Do not know |  |
| SECTION 2 - Clinical experience with MIH |  |
| Figures 1a, 1b and 1c show three patients with mild (Figure 1a), moderate (Figure 1b) and severe (Figure 1c) Incisor Molar Hypomineralization (MIH). MIH was described in 2001 as an enamel defect that affects permanent first molars and may also affect permanent incisors. The creamy-white or brownish-yellow opacities can fracture, expose dentin and facilitate the occurrence of carious lesions.  \|  \| ; **Figure 1a:** Mild MIH, only opacity \| **Figure 1b:** Moderate MIH, opacity and fracture restricted to the enamel. \| **Figure 1c:** Severe MIH, opacity, fracture exposing dentin and atypical restorations. |  |
| Considering the images and the description of MIH above, answer the following 2 questions: |  |
| **2.1- How often do you see patients with MIH in your routine as a dentist in the Brazilian Unified Health System (UHS)?** |  |
| ( ) Rarely (once every six months or less). | 0 |
| ( ) Relatively often (2 to 5 times every six months). | 1 |
| ( ) Often (once a month). | 2 |
| ( ) Very often (2 or more times a month). | 2 |
| ( ) Do not know. | 0 |
| **2.2- In your opinion, has this frequency increased during your time working as a dentist in the Brazilian Unified Health System (UHS)?** |  |
| ( ) Yes. | 2 |
| ( ) No. | 0 |
| ( ) Do not know. | 0 |
| Figure 2 shows tooth 16 of an 8-year-old patient with severe IMH, with fractures exposing dentin and a carious lesion. There are no signs or symptoms suggestive of pulp involvement. ; **Figure 2:** Tooth 16 with enamel hypomineralization, fracture exposing dentin and carious lesion. Considering this image and the description of severe IMH, answer the following 2 questions: **2.3- Considering the alternative restorative materials available at your health unit, which one would you use on this tooth?** | Same  answers in 2.3 and 2.4 = 0  Different answers in 2.3 and 2.4 = 2 |
| ( ) Amalgam. |  |
| ( ) Direct composite resin. |  |
| ( ) Self-curing hand-mixing glass ionomer cement. |  |
| ( ) Light-curing hand-mixing glass ionomer cement. |  |
| ( ) Self-curing encapsulated glass ionomer cement. |  |
| ( ) Light-curing encapsulated glass ionomer cement. |  |
| ( ) Prefabricated stainless stel crown. |  |
| ( ) Indirect restoration in resin, ceramic, porcelain or similar. |  |
| ( ) Indirect metal restoration. |  |
| ( ) Other(s). |  |
| If you answered “other(s)”, please describe the material(s) you would use: ____________________ |  |
| **2.4- If you could choose any restorative material, which would you use on this tooth?** |  |
| ( ) Amalgam. |  |
| ( ) Direct composite resin. |  |
| ( ) Self-curing hand-mixing glass ionomer cement. |  |
| ( ) Light-curing hand-mixing glass ionomer cement. |  |
| ( ) Self-curing encapsulated glass ionomer cement. |  |
| ( ) Light-curing encapsulated glass ionomer cement. |  |
| ( ) Prefabricated stainless steel crown. |  |
| ( ) Indirect restoration in resin, ceramic, porcelain or similar. |  |
| ( ) Indirect metal restoration. |  |
| ( ) Other(s). |  |
| If you answered “other(s)”, please describe the material(s) you would use: ____________________ |  |
| Figure 3 shows tooth 36 of a 7-year-old patient with severe MIH, with fractures exposing dentin and a carious lesion. There are no signs or symptoms suggestive of pulp involvement. ; **Figure 3:** Tooth 36 with enamel hypomineralization, fracture exposing dentin and carious lesion. | Same  answers in 2.5 and 2.6 = 0  Different answers in 2.5 and 2.6 = 2 |
| **2.5- Considering the alternative restorative materials available at your health unit, which one would you use on this tooth?** |  |
| ( ) Amalgam. |  |
| ( ) Direct composite resin |  |
| ( ) Self-curing hand-mixing glass ionomer cement. |  |
| ( ) Light-curing hand-mixing glass ionomer cement. |  |
| ( ) Self-curing encapsulated glass ionomer cement. |  |
| ( ) Light-curing encapsulated glass ionomer cement. |  |
| ( ) Prefabricated stainless stel crown. |  |
| ( ) Indirect restoration in resin, ceramic, porcelain or similar. |  |
| ( ) Indirect metal restoration. |  |
| ( ) Other(s). |  |
| If you answered “other(s)”, please describe the material(s) you would use: ____________________ |  |
| **2.6- If you could choose any restorative material, which would you use on this tooth?** |  |
| ( ) Amalgam. |  |
| ( ) Direct composite resin. |  |
| ( ) Self-curing powder/liquid sprayable glass ionomer cement. |  |
| ( ) Light-curing powder/liquid hand-mixing glass ionomer cement. |  |
| ( ) Self-curing encapsulated glass ionomer cement. |  |
| ( ) Light-curing encapsulated glass ionomer cement. |  |
| ( ) Prefabricated stainless steel crown. |  |
| ( ) Indirect restoration in resin, ceramic, porcelain or similar. |  |
| ( ) Indirect metal restoration. |  |
| ( ) Other(s). |  |
| If you answered “other(s)”, please describe the material(s) you would use: ____________________ |  |
| Look at the two photo sequences below:  Figures 4a to 4f show the 'Hall Technique'¹, which involves restoring decayed deciduous molars with a steel crown without preparing the tooth with a drill. |  |
|   Advantages: no need for local anesthesia, quick and inexpensive.  Disadvantages: unfavorable aesthetics, causes transient open bite as there is no occlusal lowering of the tooth.  Sequence 4g to 4i shows the Hall Technique being applied to a hypomineralized permanent first molar of a 7-year-old child.   |  |
| **2.7- Have you ever used the 'Hall Technique'?** |  |
| ( ) Yes, I've used it. |  |
| ( ) No, I've never used it. |  |
| ( ) Do not know. |  |
| **2.8 - Would you recommend the Hall Technique for cases of first permanent molars with severe MIH?** |  |
| ( ) Yes. | 2 |
| ( ) No. | 0 |
| ( ) Do not know. | 0 |
| Figure 5 shows tooth 16 of a 7-year-old patient with IMH. The tooth shows demarcated creamy-white and brownish-yellow opacities affecting the buccal, occlusal and lingual surfaces. ; **Figure 5:** Tooth 16 with enamel hypomineralization, represented by creamy-white and brownish-yellow opacities. Considering this image and the description of mild MIH, answer the following 2 questions: | Same  answers in 2.9 and 2.10 = 0  Different answers in 2.9 and 2.10 = 2 |
| **2.9- Considering the alternatives of preventive materials available in your health unit, which one would you use on this tooth?** |  |
| ( ) Periodic follow-up. |  |
| ( ) Fluoride gel application. |  |
| ( ) Application of fluoride varnish. |  |
| ( ) Application of casein amorphous calcium phosphate (CPP-ACP). |  |
| ( ) Resinous sealant. |  |
| ( ) Sealant with self-curing hand-mixing glass ionomer cement. |  |
| ( ) Sealant with light-curing hand-mixing glass ionomer cement. |  |
| ( ) Sealant with self-curing encapsulated glass ionomer cement. |  |
| ( ) Sealant with light-curing encapsulated glass ionomer cement. |  |
| ( ) Other(s). |  |
| If you answered “other(s)”, please describe the material(s) you would use: ____________________ |  |
| **2.10- If you could choose any preventive material, which one would you use on this tooth?** |  |
| ( ) Periodic follow-up. |  |
| ( ) Fluoride gel application. |  |
| ( ) Application of fluoride varnish. |  |
| ( ) Application of casein amorphous calcium phosphate (CPP-ACP). |  |
| ( ) Resinous sealant. |  |
| ( ) Sealant with self-curing hand-mixing glass ionomer cement. |  |
| ( ) Sealant with light-curing hand-mixing glass ionomer cement. |  |
| ( ) Sealant with self-curing encapsulated glass ionomer cement. |  |
| ( ) Sealant with light-curing encapsulated glass ionomer cement. |  |
| ( ) Other(s). |  |
| If you answered “other(s)”, please describe the material(s) you would use: ____________________ |  |
| During the restorative treatment of permanent first molars affected by severe MIH, indicate whether you noticed the following challenges: |  |
| **2.11- “Difficulty in dealing with the behavior of the child who tends to show more fear and anxiety than children without the same problem.”** |  |
| ( ) Yes. | 2 |
| ( ) No. | 0 |
| ( ) Do not know. | 0 |
| **2.12 - "Difficulty in obtaining proper local anesthesia due to hypersensitivity.”** |  |
| ( ) Yes. | 0 |
| ( ) No. | 0 |
| ( ) Do not know. | 2 |
| **2.13 - "Difficulty in restoring partially erupted teeth”** |  |
| ( ) Yes. | 0 |
| ( ) No. | 0 |
| ( ) Do not know. | 2 |
| **2.14- "High frequency of restoration failures.”** |  |
| ( ) Yes. | 2 |
| ( ) No. | 0 |
| ( ) Do not know. | 0 |
| **2.15- Read the following statement: “Permanent first molars affected by severe MIH cause pain, impair function and negatively impact the quality of life of children and adolescents”. From your perception, indicate how much you agree or disagree:** |  |
| ( ) Strongly disagree. | 0 |
| ( ) Disagree. | 0 |
| ( ) Neither disagree nor agree. | 1 |
| ( ) Agree. | 2 |
| ( ) I totally agree. | 2 |
| **2.16- Read the following statement: “Demarcated opacities in permanent incisors affect self-esteem and have a negative impact on the quality of life of children and adolescents”. Indicate how much you agree or disagree:** |  |
| ( ) Strongly disagree. | 0 |
| ( ) Disagree. | 0 |
| ( ) Neither disagree nor agree. | 1 |
| ( ) Agree. | 2 |
| ( ) I totally agree. | 2 |
| SECTION 3 - Continuing education in the ESF |  |
| **3.1- Have you had access to any instructional material regarding MIH, such as classes, lectures, events or other scientific content?** |  |
| ( ) Yes. | 0 |
| ( ) No. | 0 |
| ( ) Do not know. | 2 |
| **3.2- In the continuing education activities provided by the Unified Health System where you work, has the topic MIH ever been addressed?** |  |
| ( ) Yes. | 0 |
| ( ) No. | 0 |
| ( ) Do not know. | 2 |
| **3.3- Do you consider it important to address MIH in continuing education activities?** |  |
| ( ) Yes. | 0 |
| ( ) No. | 0 |
| ( ) Do not know. | 9 |
| **3.4- What topics would you like to see covered regarding MIH in continuing education activities? (you can select more than one option)** |  |
| ( ) I don't see the need to address this issue. | None = 0  1 to 3 = 2  4 or more = 4 |
| ( ) Diagnosis of MIH. |  |
| ( ) Etiology of MIH. |  |
| ( ) Preventive treatment for molars with enamel hypomineralization. |  |
| ( ) Restorative treatment for molars with enamel hypomineralization. |  |
| ( ) Aesthetic treatment of incisors with enamel hypomineralization. |  |
| ( ) Specifically the Hall Technique. |  |
| ( ) Others |  |
| **3.5- How would you like MIH to be addressed in continuing education activities (you can select more than one option)?** |  |
| ( ) No interest in the topic. |  |
| ( ) Selection of scientific articles on the subject sent by e-mail or available on an electronic storage drive. |  |
| ( ) Online theoretical and/or practical course, in real time, with the possibility of real time interaction. |  |
| ( ) Theoretical and/or practical online course available for access at any time, without interaction. |  |
| ( ) In-person theoretical and/or practical course offered in the municipality where you work as a dentist in the Brazilian Unified Health System (UHS)”. |  |
| ( ) In-person theoretical and/or practical course offered at the Faculty of Dentistry of the State University of Rio de Janeiro |  |
| **3.6- Read the following statement: “MIH should be included in the oral health records as a mandatory field.” Indicate how much you agree or disagree:** |  |
| ( ) Strongly disagree. | 0 |
| ( ) Disagree. | 0 |
| ( ) Neither disagree nor agree. | 1 |
| ( ) Agree. | 2 |
| ( ) Strongly agree. | 2 |
| **3.7- Read the following statement: “A communication channel with the university to discuss diagnosis and treatment plans for patients with MIH would contribute positively to my clinical practice as a dentist in the Brazilian Unified Health System (UHS)”. Indicate how much you agree or disagree:** |  |
| ( ) Strongly disagree. | 0 |
| ( ) Disagree. | 0 |
| ( ) Neither disagree nor agree. | 1 |
| ( ) Agree. | 2 |
| ( ) Strongly agree. | 2 |
| * The questions in sections 2 and 3, which were related to the perception of MIH as a clinical problem, were assigned with scores from 0 to 2, to measure the degree of perception using the "Scale of perception of MIH as a clinical problem by dentists". The scale ranged from 0 to 36. | |
